# Two cases of imported respiratory diphtheria in Edinburgh, Scotland, October 2019

**DOI:** 10.1017/S0950268820001028

**Published:** 2020-05-15

**Authors:** Lucy Li, Daniella Ross, Katherine Hill, Sarah Clifford, Louise Wellington, Colin Sumpter, Naomi J. Gadsby, Jennifer Crane, Karen F. Macsween, Katie L. Hopkins, Norman K. Fry, Oliver Koch, Janet Stevenson

**Affiliations:** 1 Department of Otolaryngology, NHS Lothian, Edinburgh, Scotland; 2 Regional Infectious Diseases Unit, NHS Lothian, Edinburgh, Scotland; 3 Medical Microbiology, Department of Laboratory Medicine, NHS Lothian, Edinburgh, Scotland; 4 Health Protection Team, NHS Lothian, Edinburgh, Scotland; 5 Antimicrobial Resistance and Healthcare Associated Infections (AMRHAI) Reference Unit, Public Health England, London, UK; 6 Immunisation and Countermeasures Division, Public Health England - National Infection Service, London, UK

**Keywords:** Foreign travel, Immunisation, Public health, Re-emerging infections, Respiratory diphtheria

## Abstract

We report two cases of respiratory toxigenic *Corynebacterium diphtheriae* infection in fully vaccinated UK born adults following travel to Tunisia in October 2019. Both patients were successfully treated with antibiotics and neither received diphtheria antitoxin. Contact tracing was performed following a risk assessment but no additional cases were identified. This report highlights the importance of maintaining a high index of suspicion for re-emerging infections in patients with a history of travel to high-risk areas outside Europe.

## Introduction

Diphtheria is an acute infectious disease affecting the upper and lower respiratory tracts and the skin. There are three *Corynebacterium* species that can potentially produce diphtheria toxin: *C. diphtheriae*, *C. ulcerans* and more rarely *C. pseudotuberculosis* [1].

Toxigenic *C. diphtheriae* infection is rare in the UK due to the National Immunisation Programme. Thirty-three toxigenic cases of diphtheria were identified between 2009 and 2017 in the UK, of which 18 were due to *C. diphtheriae* [1] and only three were in Scotland. The major risk factor for *C. diphtheriae* acquisition was identified as travel to an endemic area. Some countries, however, have reported a rise in the number of isolates identified as *Corynebacterium* species due to the enhanced ability to identify these bacteria using matrix-assisted laser desorption/ionization-time of flight (MALDI-TOF) systems [2]. The possibility therefore remains that some infections have remained microbiologically undiagnosed and underreported prior to the widespread introduction of MALDI-TOF.

## Case report

*Index case*: A 48-year-old UK born male was referred to a Scottish hospital on 31 October 2019 by his family doctor with a 10-day history of sore throat and dysphagia. He had returned from a holiday in Tunisia on 18 October 2019. He had previously presented to his family doctor on two occasions following the onset of symptoms. He had completed his childhood vaccinations (including diphtheria toxoid), and had a diphtheria toxoid booster 15 years previously.

On examination in the hospital, his heart rate was 98 bpm and the temperature was 37.8 °C. He had a thick white pseudomembrane extending over both pharyngeal pillars and a swollen uvula with patchy exudate. Initial blood tests showed a white cell count of 11.2 × 10^9^/l (normal range: 4–11) and C-reactive protein (CRP) of 344 mg/l (normal range: 0–5). Throat and nasopharyngeal swabs were taken. The local Health Protection Team and Public Health England (PHE) were informed, and based on epidemiological and clinical evidence the case was classed as a possible case of diphtheria. He was started on 1.2 g benzylpenicillin IV four times daily, 500 mg clarithromycin IV twice daily and 500 mg metronidazole IV three times daily. He was placed in isolation within 2 h of admission with droplet precautions.

Small numbers of *C. diphtheriae* were isolated from his throat swab on 3 November 2019. The National Reference Laboratory confirmed the identification of *C. diphtheriae* and the presence of the diphtheria toxin gene by real-time PCR [3]. The expression of diphtheria toxin was confirmed using the modified Elek immunoprecipitation test [4].

**Fig. 1.**
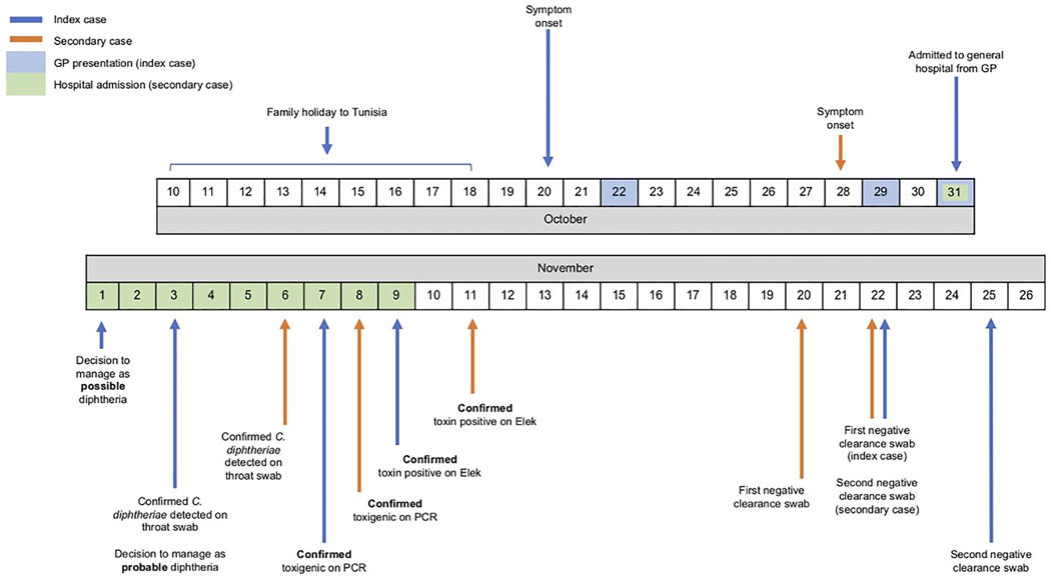
Timeline of two cases of imported respiratory diphtheria in Scotland

He improved with treatment and his pseudomembrane had largely disappeared by 8 November 2019 (Image 1). Antitoxin was not administered due to his rapid clinical improvement by the time *C. diphtheriae* was isolated. He was discharged from the Infectious Diseases Unit on 9 November 2019 with 500 mg erythromycin four times daily and 500 mg amoxicillin three times daily. A diphtheria toxoid vaccine booster was also given prior to discharge. Clearance swabs on 22 and 25 November 2019 were negative (Fig. 1).

**Figure:**
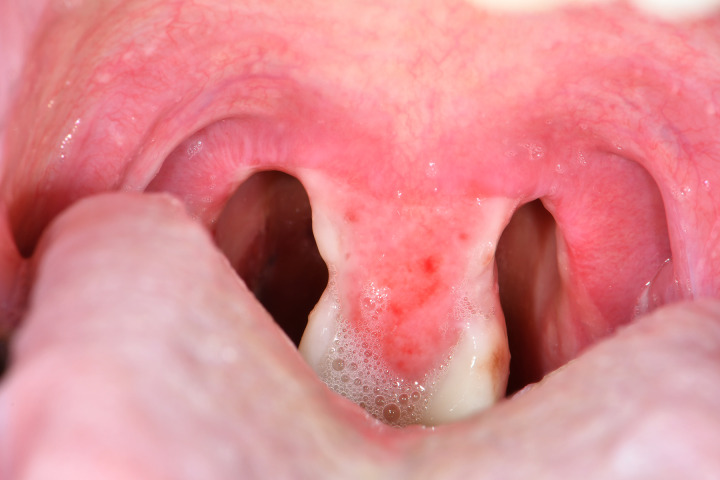

*Second case*: The second case was a 45-year-old British female who travelled with the index case to Tunisia. She reported the onset of pharyngitis from 28 October 2019. She also had a full immunisation history, but with no additional boosters. Following confirmation of *C. diphtheriae* in the index case, nasopharyngeal and throat swabs were taken and she was commenced on prophylactic oral clarithromycin 500 mg twice daily from 3 November 2019.

On 6 November 2019, when *C. diphtheriae* was isolated, she was urgently assessed at the Infectious Diseases Unit and found to have only a mildly erythematous pharynx. Her CRP was 23 mg/l. She was discharged on the same day with advice to self-isolate and to complete 14 days of clarithromycin 500 mg twice daily. Diphtheria antitoxin (DAT) was not administered. Clearance swabs on 20 and 22 November 2019 were negative (Fig. 1).

## Laboratory investigation and microbiology

Black colonies growing on Hoyle's tellurite agar (Oxoid) at 48 h, of a 72 h incubation period, from the throat swabs of both the index and the contact case [5], were further identified as *C. diphtheriae*, by MALDI-TOF mass spectrometry (MALDI Biotyper MBT Smart, Bruker), with MALDI-TOF scores of 2.29 and 2.40 for the index and the contact case, respectively. The isolates were referred to the National Reference Laboratory, PHE, London, and confirmed as *C. diphtheriae* toxin gene positive by real-time PCR and positive for toxin expression by the modified Elek test [3, 4].

The diphtheria National Reference Laboratory confirmed both isolates were *C. diphtheriae* var. mitis. Multi-locus sequence typing (MLST) profiles were derived from whole-genome sequencing using Metric-Oriented Sequence Typing [6, 7]. Alleles, allelic profiles and sequence types (STs) were determined by comparison with the *Corynebacterium diphtheriae* MLST Databases (https://pubmlst.org/cdiphtheriae/ accessioned 29/11/2019). Both isolates belonged to ST 183 (MLST allelic profiles 1, 2, 9, 7, 8, 3, 2). ST183 isolates within the MLST database are all *C. diphtheriae* var. mitis, PCR positive for diphtheria toxin and Elek positive (where recorded). Minimum inhibitory concentrations (MICs) of both penicillin and erythromycin for isolates from the index and contact case were determined by PHE's Antimicrobial Resistance and Healthcare Associated Infections (AMRHAI) Reference Unit by gradient strip testing (Table 1).

**Table 1.** In-house and antimicrobial resistance and healthcare associated infections (AMRHAI) reference unit susceptibility testing for penicillin and erythromycin

MIC (mg/l)	In-house	AMRHAI Reference Unit
Index case penicillin	0.25	0.25
Index case erythromycin	0.032	0.032
Contact case penicillin	0.5	0.5
Contact case erythromycin	0.064	0.032

## Public health control measures

An Incident Management Team was convened and contact tracing was undertaken in the community and hospital based on PHE guidelines [7]. Two children (household contacts) and one additional community contact were throat swabbed, excluded from work or school, vaccinated and prescribed antibiotics

Both cases saw several healthcare workers in the community before their diagnosis, and appropriate personal protective equipment (PPE) was not worn. Seven healthcare workers who had not worn appropriate PPE were identified as contacts (four community, three secondary care). All were screened for diphtheria carriage using the throat and nasopharyngeal swabs, given diphtheria boosters, prescribed antibiotics and excluded from work.

No *C. diphtheriae* was isolated from any of the contact's samples. Both confirmed cases were asked to self-isolate until their 14 days of antibiotic therapy was complete. Two clearance samples were sought from each case, 24 h after antibiotics had been completed, and 24 h apart [8]. These were both negative and their exclusions were lifted.

## Discussion

Primary diphtheria immunisation rates are high in Western Europe and this needs to be maintained. Our index patient had received a full diphtheria vaccination course as per UK guidelines at the time; currently, primary vaccines are given at 8, 12 and 16 weeks as part of a 6 in 1 vaccine and at 3 years 4 months as a pre-school booster, in addition to a teenage booster [9]. In Scotland, uptake of the primary course has remained consistently above 95% and the pre-school booster above 91%, although both have shown a decline in recent years [10].

For those who do not travel to diphtheria endemic countries, the UK Government considers childhood vaccinations sufficient to protect against diphtheria outbreaks with no further adult boosters. However, outside of natural exposure, immunity is known to wane over time and we do not know the effectiveness of a vaccination course beyond 39 years [11, 12]. Seroepidemiological surveys of non-endemic countries have demonstrated seropositivity well below that required for immunity in older age groups [12]. Partial immunity is a possible explanation for the relatively mild presenting symptoms in our index patient and routine teenage or regular adult boosters have been recommended in some countries to counter this, including the United States of America [11, 13], however, there is currently no evidence this reduces the incidence of diphtheria at a population level [14].

A diphtheria vaccine booster is recommended for those travelling to high-risk countries for more than 10 years since their last dose [15]. Tunisia is not currently included as a high-risk country and revising travel advice may be timely [15]. According to WHO, there have not been any cases of diphtheria in Tunisia since 1993 and vaccination rates have been over 90% since 1988 [16]. However, there have been no data published since 2017. Because of the recent increase of migration into Tunisia from higher-risk diphtheria countries, the risk of diphtheria in Tunisia may be increasing [16, 17]. Data from the *C. diphtheriae* MLST databases shows that ST183 isolates are predominantly from Algeria. As Algeria borders Tunisia, this would support the hypothesis of the acquisition of this infection in Tunisia. The two cases had minimal exposure with people beyond their holiday resort and airport with no other known high-risk contact or activity.

Interpretation of the penicillin and erythromycin MICs is problematic given that EUCAST guidelines (v. 10.0) indicate that the current *Corynebacterium* spp. benzylpenicillin breakpoint (*R* > 0.125 mg/l) is not suitable for *C. diphtheriae* and breakpoints for erythromycin are under preparation. However, penicillin MICs of 0.25–0.5 mg/l are unexceptional when compared to penicillin MICs observed for *C. diphtheriae* referred to the AMRHAI Reference Unit (K.L. Hopkins, 30 December 2019, personal communication, unreferenced). Dual antibiotic therapy was, therefore given due to these concerns regarding potential antibiotic resistance.

Diphtheria antitoxin (DAT) is an equine hyperimmune antiserum used to inactivate the diphtheria toxin [18] with a high reported mortality benefit if given early in diagnosis [19]. It has a significant risk of hypersensitivity reactions, including anaphylaxis, and is given cautiously [20]. Our index case improved rapidly on intravenous antibiotics, and given the initial diagnostic uncertainty, DAT was not given. The secondary case as well and did not require DAT.

The early isolation of the index case reduced the number of exposed healthcare contacts requiring to follow up and even fewer healthcare contacts would have required public health follow up if appropriate PPE had been worn during clinical investigations.

We recommend an early multi-disciplinary discussion of possible cases of rare imported infections with public health implications as classification is open to interpretation, particularly in the case of rarely seen re-emerging infections.

## Conclusion

Respiratory diphtheria is now rare in the UK, resulting in the limited first-hand experience for frontline clinicians. Maintaining a high index of suspicion is essential, especially if a case has travelled outside Europe and helps to ensure successful clinical and public health management of a case.
